# A Culturally Adapted Cognitive Behavioral Internet-Delivered Intervention for Depressive Symptoms: Randomized Controlled Trial

**DOI:** 10.2196/13392

**Published:** 2020-01-31

**Authors:** Alicia Salamanca-Sanabria, Derek Richards, Ladislav Timulak, Sarah Connell, Monica Mojica Perilla, Yamilena Parra-Villa, Leonidas Castro-Camacho

**Affiliations:** 1 Trinity College Dublin School of Psychology E-mental Health Research Group Dublin Ireland; 2 SilverCloud Health Clinical Research & Innovation Dublin Ireland; 3 Universidad Autonoma de Bucaramanga School of Psychology Bucaramanga Colombia; 4 Universidad de Los Andes School of Psychology Bogota Colombia

**Keywords:** culture, internet, cognitive behavioral therapy, depressive symptoms, students, South America

## Abstract

**Background:**

Internet-delivered treatments for depressive symptoms have proved to be successful in high-income Western countries. There may be potential for implementing such treatments in low- and middle-income countries such as Colombia, where access to mental health services is limited.

**Objective:**

The objective of this study was to assess the efficacy of a culturally adapted cognitive behavioral internet-delivered treatment for college students with depressive symptoms in Colombia.

**Methods:**

This was a randomized controlled trial with a 3-month follow-up. The program comprised seven modules. A total of 214 Colombian college students were recruited. They were assessed and randomly assigned to either the treatment group (n=107) or a waiting list (WL) control group (n=107). Participants received weekly support from a trained supporter. The primary outcome was symptoms of depression, as measured by the Patient Health Questionnaire - 9, and the secondary outcomes were anxiety symptoms assessed by the Generalized Anxiety Disorder questionnaire - 7. Other measures, including satisfaction with treatment, were evaluated after 7 weeks.

**Results:**

Research attrition and treatment dropouts were high in this study. On average, 7.6 sessions were completed per user. The mean time spent on the program was 3 hours and 18 min. The linear mixed model (LMM) showed significant effects after treatment (*t*_197.54_=−5.189; *P*<.001) for the treatment group, and these effects were maintained at the 3-month follow-up (*t*_39.62_=4.668; *P*<.001). Within-group results for the treatment group yielded a large effect size post treatment (*d*=1.44; *P*<.001), and this was maintained at the 3-month follow-up (*d*=1.81; *P*<.001). In addition, the LMM showed significant differences between the groups (*t*_197.54_=−5.189; *P*<.001). The results showed a large effect size between the groups (*d*=0.91; *P*<.001). In the treatment group, 76.0% (16/107) achieved a reliable change, compared with 32.0% (17/107) in the WL control group. The difference between groups was statistically significant (X^2^_2_=10.5; *P*=.001).

**Conclusions:**

This study was the first contribution to investigating the potential impact of a culturally adapted internet-delivered treatment on depressive symptoms for college students as compared with a WL control group in South America. Future research should focus on identifying variables associated both with premature dropout and treatment withdrawal at follow-up.

**Trial Registration:**

ClinicalTrials.gov NCT03062215; https://clinicaltrials.gov/ct2/show/NCT03062215

## Introduction

High rates of prevalence of depression have been found worldwide and in various cultural groups [1-5], with an upward trend in prevalence in low- and middle-income countries (LMICs) [6]. In South American countries such as Colombia, high prevalence rates are reported, which are similar to the prevalence rates found in high-income countries (HICs) [3,4]. In Colombia, the 12-month prevalence rate has been estimated to be between 6.6% and 10% [7].

Studies have shown that Colombia reports the second highest prevalence of major depression in Latin America after Brazil [3,8]. An epidemiological report on the prevalence of mood disorders in LMICs shows that Colombia ranks first (6.9%), compared with India (5.5%), Iraq (4.1%), Nigeria (1.2%), Beijing (2.2%), and Shenzhen (4.8%) [9]. Recently, the Pan American Health Organization and World Health Organization [10] reported that most of the South American countries have higher depression disability (7.7%), specifically Paraguay (9.4%), Brazil (9.3%), Peru (8.6%), and Colombia (8.2%). The Colombian National Mental Health Survey [11] estimated a point prevalence of mild to moderate depressive symptoms in 15.6% and severe depressive symptoms in 4.2% of adults. In addition, a report [12] shows that moderate depression among women is 70.4% compared with that among men (29.6%). Depressive symptoms can go undiagnosed and untreated, and the absence of treatment may be associated with the development of a depressive disorder [13].

Depressive symptoms are commonly reported in general populations and among college students [14-16]. Cross-cultural studies show high rates of depressive symptoms among students [17,18]. Depressive disorders are more frequent among college students in comparison with the general population [19]. For instance, a systematic review showed a 30.6% weighted mean prevalence of depression symptoms in college students from Europe, the United States, Canada, Egypt, Turkey, Korea, Lebanon, and China [20]. These rates are comparable with those from Latin American countries [21-24]. In Colombia, a study reported high prevalence of depressive symptoms (30%) in students [25]. Another recent study showed 36.2% prevalence of depression symptoms in a college sample [26]. A recent report from Colombia showed that people with university studies presented with higher levels of depression (61.6%) compared to those without a higher education (45.2 %) [12]. Several variables have been associated with the vulnerability of college students to depression: changes in lifestyle related to poor sleep habits, eating disorders, economic stressors, and family problems [20,27].

Such a high prevalence warrants implementation of interventions for depression in Colombia. However, as in many other LMICs, there are barriers for accessing mental health treatments, such as cost and coverage, that prevent people from accessing the treatments they need [11,26]. In 2016, the World Mental Health survey reported that only 6.7% of college students received treatment for their mental health disorder in LMICs [28]. Furthermore, the population with mental health problems does not have an adequate insurance coverage [11,29]. The Colombian National Mental Health Survey in 2015 showed that about 50% of the population reported that personal stigma is one of the principal reasons for not accessing mental health services, followed by geographical location and limited service availability [11].

Despite the necessity to implement psychological interventions for depression in Latin America, there are only a few studies in this field [30], more so because treatments are mostly pharmacological, and evidence-based psychological treatments are rarely used. Furthermore, most of the treatments in LMICs are implemented without considering the cultural context of the clients; very little research has investigated culturally adapted treatments [31,32], whereas there is even less research on internet-delivered treatments [33,34] that may be a suitable alternative to make evidence-based treatments available, especially when nearly 56% of the population in Colombia has access to the internet [35].

Depressive symptoms can be effectively treated with psychotherapy and pharmacotherapy [36-39]. Psychological treatments for depression have shown better outcomes than a waiting list (WL) or placebo, and combined treatment is more effective than pharmacotherapy alone [38,40]. Research has found that psychological treatments can enhance the positive effect and decrease the negative effect in depression, contributing to better outcomes [41]. A systematic review and a meta-analysis have shown that cognitive behavioral therapy (CBT) had been extensively researched and was demonstrated to be effective for depression [42,43].

Low-intensity internet-delivered treatment is usually based on cognitive behavioral principles. It involves complete or partial guidance and uses CBT techniques that are typically used in face-to-face therapy, for instance, reading passages, listening to audio files, seeing pictures, and watching animations and videos [44,45]. This type of intervention is highly structured, and it involves psychoeducation, activities, and supplementary resources such as a supporter contact via asynchronous message [46].

Internet-delivered interventions have an established empirical base for major [47] and subthreshold [48,49] depression. They are also used as maintenance treatments [50-52]. For instance, a systematic review of computer-based interventions for depression, with a supporter, found a medium-to-large (0.58-0*.*76) and a small-to-medium (0.25-0.36) effect size when the intervention was delivered as a stand-alone self-help treatment for the reduction of depressive symptoms, both compared with usual care [51].

Internet-delivered interventions for depression are available in many languages. Research with Spanish language versions [53] reported positive results of the effectiveness of a CBT-based internet-delivered program for depression. A recent 4-year descriptive, naturalistic study monitoring a Web-based CBT treatment developed and researched in Mexico indicated that the intervention was useful for depressive symptoms [54]. In addition, a feasibility study in Chile found a Web-based treatment to be beneficial, acceptable, and feasible [55].

For the most part, there is availability of psychological treatments and mental health services in HICs. In addition, research evidence of internet-delivered interventions and their implementation have been mainly developed in HICs [56], whereas in LMICs, most people who need mental health services do not receive any—this is known as the *mental health gap*. For instance, the World Health Organization [57] has estimated that between 76% and 85% of the people with severe mental health problems receive no treatment in LMICs. Therefore, internet-delivered interventions may be a valuable resource to reduce the treatment gap in LMICs, such as Colombia, thus ameliorating social health inequalities in these regions [58]. In addition, there is little knowledge on internet-based interventions for depression emerging in such countries [34]. A recent systematic review found that only three articles reported the results of randomized controlled trials (RCTs) in internet-delivered interventions for mental health conditions in LMICs [33].

This study aimed at evaluating the efficacy of a culturally adapted internet-delivered CBT (iCBT), *Yo puedo sentirme bien/I can feel better* program, for college students with depressive symptoms. This study is the first contribution to investigating the efficacy of an iCBT intervention in Colombia.

## Methods

### Study Design

The study used a randomized control design (trial registration: ClinicalTrials.gov NCT03062215) to examine the efficacy of the culturally adapted intervention in which participants were randomly assigned to two groups: (1) a culturally adapted iCBT group and (2) a WL control group.

### Participants

College students from two cities and universities in Colombia were included in this study. Both undergraduate- and graduate-level students from any school at a university in Bogota City and students of psychology, medicine, nursing, and education from a university in Bucaramanga City in Colombia were eligible to participate, and they were selected according to the criteria described in Textboxes 1 and 2.

**Inclusion criteria.**

- Age ≥18 years
- Mild to moderately severe depressive symptoms determined by the Patient Health Questionnaire - 9 scores of 10-19

**Exclusion criteria.**

- Severe depressive symptoms: score of >19 on the Patient Health Questionnaire - 9
- Suicidal ideation or intent: score of ≥2 on question 9 of the Patient Health Questionnaire - 9
- Psychosis
- Currently in psychological treatment for depression
- On medication for <1 month
- Alcohol or drug misuse
- Previous diagnosis of an organic mental health disorder
- Depression preceding or coinciding with a diagnosed medical condition

### User Recruitment

An email with information about the study and a link to access to the treatment was sent to all college students (undergraduates and postgraduates) in Bogota university and to undergraduate students studying medicine, psychology, and education in Bucaramanga university. Potential participants were able to visit a website to receive information about the study, participation criteria, treatment, and how to get in contact to proceed with the study. Recruitment took place between August 2016 and January 2017.

### Procedure

#### Overview

Once participants read the study information, informed consent was obtained from each user before screening and randomization. Through the SilverCloud platform, participants were instructed to type their name on the informed consent page to indicate that they had read and understood the study information and agreed to participate in the study. Thereafter, participants completed measures for screening purposes, including the Patient Health Questionnaire - 9 (PHQ-9), Sociodemographic and Clinical History Questionnaire, and Generalized Anxiety Disorder - 7 (GAD-7) questionnaire. Thereafter, participants who were eligible for the study (Textbox 1) were randomized through computer algorithms and assigned to one of two groups: the iCBT treatment and WL control groups.

Participants assigned to the active treatment group started the internet-delivered treatment immediately for 7 weeks, whereas treatment for the WL participants started after 7 weeks. Individuals not meeting the inclusion criteria at baseline assessment were referred to other appropriate sources of face-to-face support at the student counseling service in their respective universities. Once the students completed the questionnaires, they received immediate feedback on their results. If exclusion from the study was necessary, information and advice to seek counseling services at their college was provided to the student. In addition, the counseling service manager received an automatic email with information of the excluded students, their questionnaires (ie, Sociodemographic and Clinical History Questionnaire, PHQ-9, and GAD-7), and their scores. Participants received emails and calls from the research team at the posttreatment and 3-month follow-up time points to encourage them to complete their assigned measures.

#### Randomization

**Figure 1:**
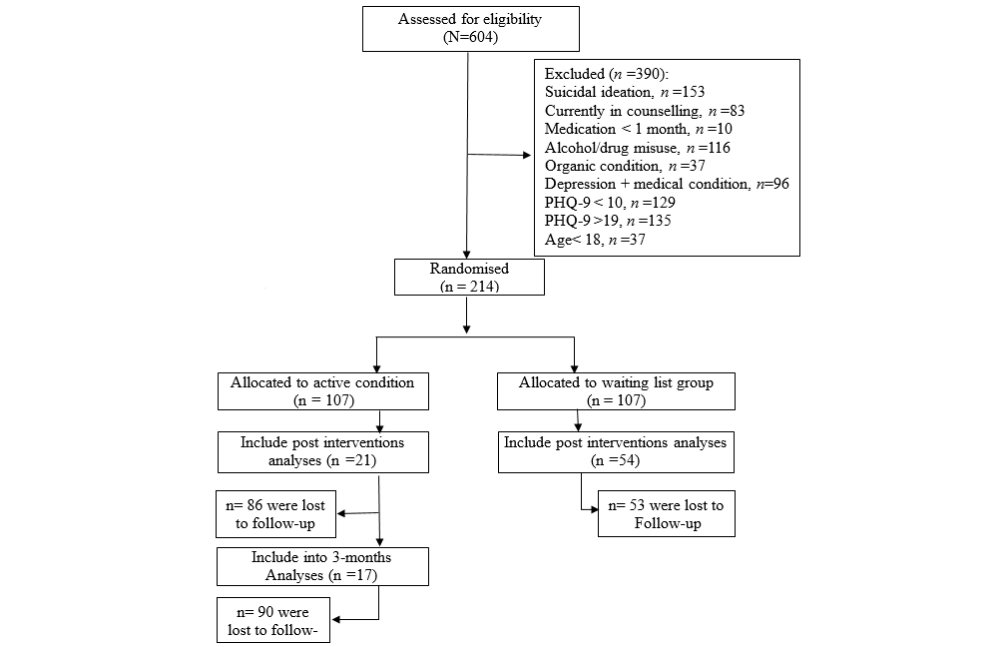
Study participants flowchart—Consolidated Standards of Reporting Trials diagram. PHQ-9: Patient Health Questionnaire - 9.

After baseline screening, eligible participants were randomized and informed immediately about their group assignment. Randomization was handled by a computer algorithm administered by a person independent of the researchers. Figure 1 shows the Consolidated Standards of Reporting Trials (CONSORT) participant flow through the trial. 

#### Intervention

The Colombian iCBT program for depression assigned as treatment in this study (*Yo puedo sentirme bien/*I can feel better) is a modified version of the clinically efficacious *Space from Depression* program [59] used in Ireland and the United Kingdom. The name *Yo puedo sentirme bien* (*I can feel better* in English) was selected as a culturally appropriate descriptor for improving mood to avoid the negative connotations related to depression in Colombian culture.

The cultural adaptation that underwent several steps is described in a separate study [60]. The adaptation involved three distinct phases based on an integrative approach proposed by the author (AS). In the first phase, it involved a top-down cultural sensitivity framework, developed by the principal researcher (AS). The preliminary adaptation included work done by professional translators and an independent video company in Colombia. The first phase in the adaptation comprised a translation of the program from English to Spanish and the incorporation of cultural expressions, examples, and personal stories into the program. The second phase comprised an assessment of the preliminary program through the Cultural Relevance Questionnaire (CRQ) that was designed specifically for this study. The CRQ is based on cultural sensitivity and ecological validity theory [61] and principles from cross-cultural assessment research [60,62]. The CRQ has two sections: (1) a general assessment of the program and (2) an assessment of each module, including feedback from college students and psychology experts. The third phase involved further development of cultural incorporations into the program based on this feedback [60].

Both *Yo puedo sentirme bien* (the Colombian iCBT program used as treatment in this study) and *Space from Depression* (the original program from which it was culturally and linguistically adapted) comprise seven modules of CBT (Table 1) [63]. The treatment includes self-monitoring, behavioral activation, cognitive restructuring, and challenging core beliefs. All modules have the same structure and format, comprising quizzes, videos, educational content, and activities with homework suggestions and a module review page (Multimedia Appendices 1 and 2). Moreover, users have a supporter who provides weekly feedback asynchronously [59].

The participants accessed the intervention for free during the time of the study. They also had access to the program without a supporter for a year after the study if they wanted to use it.

**Table 1:** Yo puedo sentirme bien/Space from Depression program description.

Module			Brief description
Getting started			Outlines the basic premise of cognitive behavioral therapy, provides information about depression, and introduces some of the key ideas of *Space from Depression*; users are encouraged to begin to chart their own current difficulties with depression
**Tune in**			
	I: Getting to grips with mood	The focus in this module is on mood monitoring and emotional literacy; users can explore different aspects of emotions, physical reactions, action and inaction, and how they are related	
	II: Spotting thoughts	This module focuses on noting and tracking thoughts; users can explore the connection between their cognitions and mood and record them graphically	
**Change it**			
	I: Boosting behavior	This module focuses on behavioral change as a way to improve mood; ideas about behavioral activation are included, and users can plan and record activities and chart their relationship with their mood	
	II: Challenge your thoughts	This module supports users to challenge distorted or overly negative thinking patterns, with thought records as well as helpful coping thoughts	
	III: Core beliefs	This module outlines the role that deeply held core beliefs could play in mood and depression; users can use a range of interactive activities to identify, challenge, and balance any unhelpful core beliefs	
Bringing it all together			In this final module, users are encouraged to bring together all the skills and ideas they have gathered so far, note their personal warning signs, and make a plan for staying well

#### Wait List Control Group

Participants in the WL control group received treatment only after 7 weeks; therefore, this group received no treatment in the first 7 weeks of the study.

#### Supporters

Each of the 214 participants were assigned a supporter, who was a postgraduate student in clinical psychology with experience delivering CBT for adults. A total of 10 supporters were trained by the principal researcher (AS) to use the *Yo puedo sentirme bien*/*I can feel better* platform and program before starting their role as a supporter, and they were supervised by an experienced clinical psychologist at the university.

Each supporter was assigned users. Supporters provided asynchronous postsession feedback of 10-15 min per participant per week through the platform. The role of the supporter was to motivate and provide feedback to the users. The feedback was focused on improving adherence to the interventions and encouraging the application of CBT activities in the program. They scheduled the feedback at specific times, once each week for a period of 7 weeks. This schedule of support replicated what had already been implemented with the program in the Irish RCT*.* The messages explain how to work through the program; the supporters also suggested modules and activities to the users. All the messages were personalized, depending on the user’s necessities (the content used or specific questions or comments the user made to the supporter through the platform). Further, 7 weekly automated alert messages were sent by email to all the participants, indicating that they had received a new review from their supporter.

### Assessments

Through the SilverCloud platform, the assessments were completed by the participants. For the efficacy trial, participants were assessed at baseline, posttreatment, and 3-month follow-up; details of the measures are described in Table 2.

Participants were assessed at baseline using the PHQ-9, Sociodemographic and Clinical History Questionnaire, and GAD-7. At the beginning of each session, participants were asked to reflect on their previous session and complete the Helpful Aspects of Treatment Form, a qualitative measure, the results of which are not presented in this paper. PHQ-9 and GAD-7 were completed at week 7 and follow-up was completed at week 20 (3 months). A Satisfaction With Treatment (SAT) questionnaire was completed after week 7.

**Table 2:** Measures used (study: efficacy trial—screening and primary and secondary outcomes).

Measure	Assessed variable	Time of assessment
Patient Health Questionnaire - 9	Depression symptoms	Baseline, posttreatment, and follow-up
Generalized Anxiety Disorder - 7	Anxiety symptoms	Baseline, posttreatment, and follow-up
Sociodemographic and Clinical History Questionnaire	Gender, age, marital status, education, occupation, socioeconomic status, and clinical history	Baseline
Helpful and Hindering Aspects of Treatment>	Helpful and hindering aspects of treatment	After each session
Satisfaction	Satisfaction with the therapy	Posttreatment

#### Screening Measure

##### Sociodemographic Information and Clinical History Questionnaire

This instrument was developed on the basis of a previous version [64]. It collects data on the participants, such as the length of time one is experiencing depression symptoms; the participant’s experience of counseling/therapy and medication for depression; and whether one has had a previous diagnosis of an organic mental health disorder or serious mental health disorders such as schizophrenia, psychosis, and bipolar disorder. In addition, it contains items related to comorbidity of depression, such as alcohol and drug misuse, or any recent medical diagnosis.

#### Outcome Measures

##### Primary Outcome: Patient Health Questionnaire - 9

The PHQ-9 [65] is a 9-item self-report measure that assesses the nine depression symptoms from the Diagnostic and Statistical Manual of Mental Disorders, fourth edition (DSM-IV), depression criteria. Each item is scored on a 4-point scale (0-3), and scores range from 0 to 27. The score can be used to describe a patient’s symptoms in one of the five categories: none (0-4), mild (5-9), moderate (10-14), moderately severe (15-19), and severe (20-27). The PHQ-9 has been shown to have good internal reliability (Cronbach alpha=0.86-0.89) [66]. The PHQ-9 has been translated into Spanish, and a Colombian version will be used in this study. The Spanish version of PHQ-9 has also demonstrated good reliability among Latinos in the United States (alpha=.85) [67,68]. In this study, the internal consistency of the PHQ-9 in the sample was .87.

##### Secondary Outcome: General Anxiety Disorder - 7

The GAD-7 [69] comprises seven items measuring symptoms and severity of anxiety based on the DSM-IV diagnostic criteria. It has shown good internal reliability (alpha=.92) [70]. It has been culturally adapted into Spanish [71] and is available in a Colombian Spanish version. In this study, the internal consistency of the GAD-7 in the sample was .88.

#### Other Measures

##### Posttreatment: Satisfaction With Treatment

At posttreatment, participants were asked to complete the SAT questionnaire [72]. It asks users about positive and negative experiences with the internet-delivered treatment. It contains two questions asking participants to describe what they most and least liked about the Web-based treatment.

### Data Analysis

All participants’ data were included, irrespective of treatment compliance. Descriptive statistics (Chi-square and *t* tests) were used to analyze sociodemographic and clinical variables at the baseline among the groups (eg, gender and age) [73].

The effect of treatment on PHQ-9 and GAD-7 severity scores was evaluated separately for each measure by using the repeated-measures linear mixed-effects models fit with restricted maximum likelihood in the R package lme4 [74]. The core computational algorithms are implemented using the “Eigen” C++ library for numerical linear algebra and “RcppEigen glue” in the R (general public license ≥ version 2.0) [75]. Linear mixed-effects models are appropriate for analysis of longitudinal data where repeated measurements are taken from the same subjects over time and are particularly robust to missing data. All participants who provided posttreatment measures or 3-month follow-up measures, irrespective of treatment compliance, were included. Linear mixed model (LMM) fit by the restricted maximum likelihood (REML) *t* test using Satterthwaite method was employed to estimate covariance matrix, including fixed effects for time [74,76]. The REML approach is a particular form of maximum likelihood estimation that does not base estimates on a maximum likelihood fit of all the information, but instead uses a likelihood function calculated from a transformed set of data. For each measure, total scores were modeled using fixed effects of time, treatment group and the interaction between time and treatment group, and a random effect of individual/identification. The significance of each fixed effect was evaluated with *t* tests using the Satterthwaite degrees of freedom method [77]. Post hoc pairwise comparison of means estimated from the models were performed using the R package. All reported *P* values are two-tailed, with significant levels at *P*<.01, *P*<.01, and *P*<.05. The magnitude of the treatment effect within and between the two groups was assessed using the Cohen *d* statistic [73]. Cohen describes an effect size of 0.2 as small, 0.5 as medium, and 0.8 as large [78].

Analyses were conducted to assess how many participants achieved clinically significant changes at the end of the intervention and at follow-up. The assessments were made through a comparison of pretreatment scores with posttreatment and follow-up scores on the outcome measures PHQ-9 and GAD-7. Reliable change was assessed using the Jacobson and Truax reliable change criteria. Internal consistency, measured with Cronbach alpha, was used as the reliability estimate, and it was high for both the PHQ-9 (alpha=.89) and GAD-7 (alpha=.88). Change on either measure was considered reliable if it exceeded the resulting reliable change index, which was calculated to be 3.98 for the PHQ-9 and 3.61 for the GAD-7. Reliable recovery criteria were calculated as the combination of the reliable change index (RCI) and cutoff score and the percentage of participants who achieved a posttreatment score of ≤10 on the PHQ-9 and ≤8 on the GAD-7.

Descriptive statistics were used to analyze the quantitative data from the SAT questions, and the qualitative responses from this questionnaire were analyzed using thematic analysis. The categorization of data followed that identified in the previous research [79].

### Ethics

The research project and all related materials were submitted and approved by the appropriate university ethics committees in Colombia. Participants were provided with the study information including the aims and objectives and provided informed consent. Participants meeting exclusion criteria were referred to other appropriate sources of support at the universities. The study protocol, information on the study, informed consent, and related materials were submitted and approved by the ethics committee of the two universities.

## Results

### Baseline Characteristics

Descriptive statistics revealed that at postrandomization, there were no significant differences in the sample between the iCBT and WL control groups on any variables. Table 3 shows details of the characteristics of the sample. The mean age was 22.15 (SD 4.74) years. All the participants were full-time students. The majority of the college student sample was from health sciences (86/214, 40.2%), followed by social sciences (62/214, 29.0%), and engineering (37/214, 17.3%).

There was no significant difference between the proportions of participants who reported a previous diagnosis of depression in the control group versus the treatment group. The majority of the participants reported depressive symptoms of between 1 and 2 years (35.0%, 75/214). Of the participants, 21.9% (47/214) reported to have had counseling/psychotherapy for depression in the past. Finally, the sample reported confidence in their use of information technology (IT), with 33.6% (72/214) of participants reporting feeling confident. Of the participants, 40.6% (87/214) reported feeling not so confident using technology.

**Table 3:** Demographic and clinical variables.

Variable			Total (N=214), n (%)		Treatment (N=107), n (%)		Control group (N=107), n (%)		*P* value	
**Gender**									**.56**	
	Female	153 (71)		74 (69.2)		79 (73.8)				
	Male	61 (28.5)		33 (30.8)		28 (26.2)				
**Age (years)**										
	Mean (SD)	22.15 (4.7)		22.24 (5.4)		22.06 (3.9)		.98		
	Range	N/A^a^		18-52		18-37		—		
**Education subject**									**.81**	
	Health sciences	86 (40.2)		46 (43.0)		40 (37.4)				
	Social sciences	62 (29.0)		31 (29.0)		31 (29.0)				
	Engineering	37 (17.3)		17 (15.9)		20 (18.7)				
	Postgraduate subjects	29 (13.6)		13 (12.1)		16 (15.0)				
**Time with symptoms**									**.31**	
	<6 months	56 (26.1)		28 (26.2)		28 (26.2)				
	1-2 years	75 (35.0)		42 (39.3)		33 (30.8)				
	2-5 years	43 (20.0)		22 (20.6)		21 (19.6)				
	>5 years	40 (18.6)		15 (14.0)		25 (23.4)				
**Previous therapy for depression**									**.32**	
	Yes	47 (21.9)		20 (18.7)		27 (25.2)				
	No	167 (78.0)		87 (81.3)		80 (74.8)				
**Previous medication for depression**									**.72**	
	Yes	37 (17.2)		20 (18.7)		17 (15.9)				
	No	177 (82.7)		87 (81.3)		90 (84.1)				
**Current medication for depression**									**>.99**	
	Yes	7 (3.2)		3 (18.7)		4 (15.9)				
	No	207 (96.7)		104 (81.3)		103 (84.1)				
**Information technology–related confidence**									**.02**	
	Very confident	6 (2.8)		2 (1.9)		4 (3.7)				
	Confident	72 (33.6)		35 (32.7)		37 (34.6)				
	Average	4 (1.8)		2 (1.9)		2 (1.9)				
	Mildly confident	72 (33.6)		52 (23.4)		20 (18.7)				
	Not confident	87 (40.6)		43 (40.2)		44 (41.1)				

^a^Not applicable.

### Treatment Response Rate

**Figure 2:**
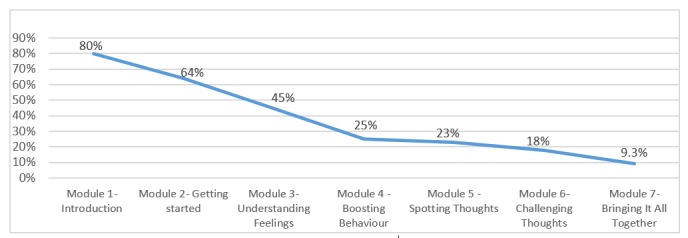
Percentage of users accessing modules over time.

Participants were offered 7 modules to complete. Similar to the original efficacy trial, although they were instructed to complete each of the 7 modules, the choice, pace, and control over the direction and dose of their engagement was entirely up to them [59]. Of the 107 participants randomized to the immediate treatment group, 80% (86/107) began module 1, and 9.3% (10/107) completed all modules. Figure 2 represents the treatment response rate over time.

A session in a Web-based intervention is defined as an instance when a client logged into the system [59]. Session time estimation is not exact because users may be interrupted or take breaks within a session and may not formally log out of the system. Regarding the treatment group, the total number of sessions completed was 734, with an average of 7.6 sessions completed per user. The mean time spent on the program was 3 hours and 18 min.

### Research Data Attrition

A total of 604 participants were screened, of which 390 were excluded based on the established criteria. A significant number of participants were excluded because of suicidal ideation (153/604, 25.3%) and misuse of drugs or alcohol (116/604, 19.2%). At sign-up, there were a total of 107 participants assigned to the treatment group and 107 assigned to the WL control group. See CONSORT flow in Figure 1.

The Little´s Missing Completely at Random (MCAR) test revealed a nonsignificant result (X^2^_4_=1.28; *P*=.86), indicating that the data were missing completely at random [80]. The percentage of participants who completed research measures at postintervention was 19.6% (21/107) and the 3-month follow-up was 16.8% (18/107). In the WL group, the response was higher, with 50.4% (54/107) of participants present at the postintervention assessment. The WL control group was not followed up beyond this point, as they entered treatment.

### Intention-To-Treat Analysis

Owing to the very high percentage (86/107, 80.3%) of missing data in the sample, we decided not to follow the protocol data analysis strategy [63]. Instead, we modified our approach to include only those participants who provided posttreatment measures (21/107, 19.6%) or the 3-month follow-up (18/107, 16.8%) measures.

#### Patient Health Questionnaire - 9

The iCBT and WL control group samples were compared using LMMs, adjusting for depression and anxiety scores in the model. LMM including fixed effects for time showed significant effects after treatment (*t*_38.23_=−5.079; *P*≤.001) within the iCBT treatment group. The effects were also maintained at the 3-month follow-up (*t*_39.62_=−4.668; *P*≤.001) for the treatment group (see Table 4). Also, LMM shows significant differences between the groups (*t*_197.54_=−5.189; *P*≤.001) in favor of the treatment group.

**Figure 3:**
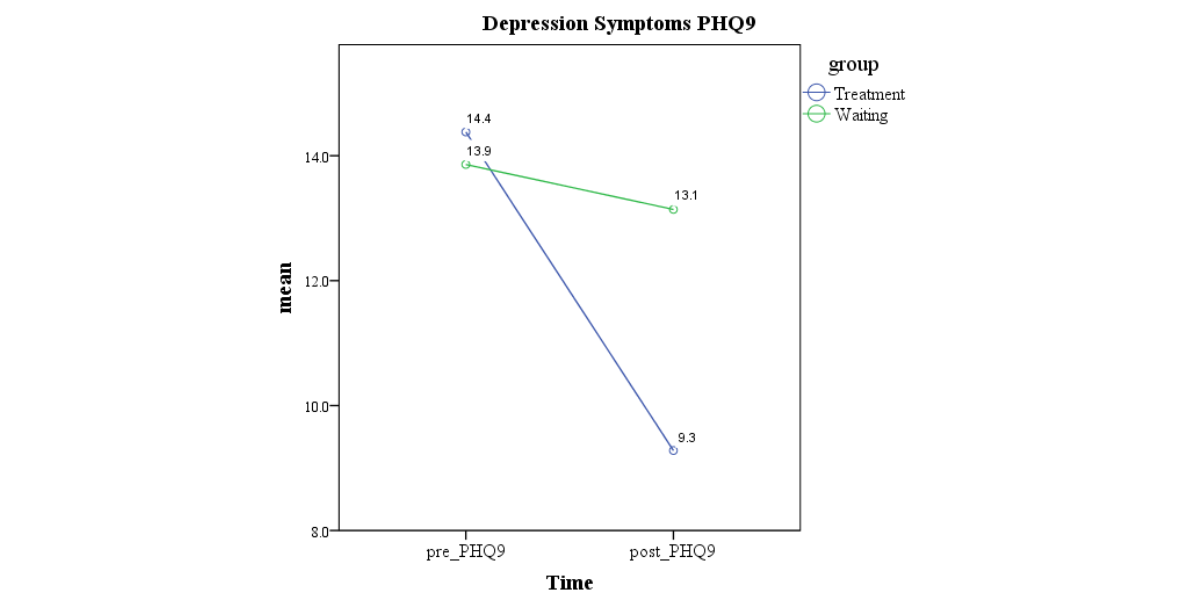
Changes in depression symptoms pre- to postintervention. PHQ-9: Patient Health Questionnaire - 9.

The within-group results for the treatment group yielded a large effect size after treatment (*d=*1.44; *P*≤.001), and this was maintained at the 3-month follow-up (*d=*1.81; *P*≤.001). Similarly, the results showed a large effect size between groups (*d=*0.91; *P*≤.001). Figure 3 provides a graphical representation of the changes in depressive symptoms between groups pre- and posttreatment.

**Table 4:** Descriptive data for the Patient Health Questionnaire - 9 and Generalized Anxiety Disorder - 7 by group over time (intention to treat).

Outcome measures		Treatment							Follow-up				
		Baseline		Pretreatment		Posttreatment		Within-group effect size (95% CI)	Baseline		3-month follow-up		Effect size (95% CI)
		*t* (*df*)	*P* value^a^	n	Mean (SD)	n	Mean (SD)		*t* (*df*)	*P* value^b^	n	mean (SD)	
**Patient Health Questionnaire - 9**													
	Treatment group	−5.079 (38.23)	≤.000	107	14.22 (2.81)	21	8.33 (5.71)	1.44 (0.38 to 2.49)	−4.668 (39.62)	≤.000	17	8.41 (5.98)	1.81 (0.69 to 2.92)
	Waiting list group	N/A^c^	N/A	107	13.82 (2.93)	54	13.09 (5.06)	0.20 (−0.78 to 0.58)	N/A	N/A	N/A	N/A	N/A
**Generalized Anxiety Disorder - 7**													
	Treatment group	−2.632 (37.83)	≤.012	107	10.56 (4.380)	21	7.19 (4.79)	0.73 (0.08 to 1.37)	−2.486 (38.88)	≤.017	17	7.83 (6.23)	0.52 (−0.17 to 1.21)
	Waiting list group	N/A	N/A						N/A	N/A	N/A	N/A	N/A

^a^Compared to posttreatment.

^b^Compared to the 3-month follow-up.

^c^Not available.

#### Generalized Anxiety Disorder - 7

Similarly, the iCBT group and WL control group samples were compared using LMM. Within the iCBT group, time point was a significant predictor of the GAD-7 score, with a significant effect from baseline to posttreatment (*t*_37.83_=−2.632; *P*≤.012) and the 3-month follow-up (*t*_38.8_=−2486; *P*≤.017; Table 4) within the treatment group. The interaction effect was also significant, representing significant differences in the GAD-7 score change observed from baseline to posttreatment between the groups (*t*_103.53_=−2.229; *P*≤.03) in favor of the treatment group.

**Figure 4:**
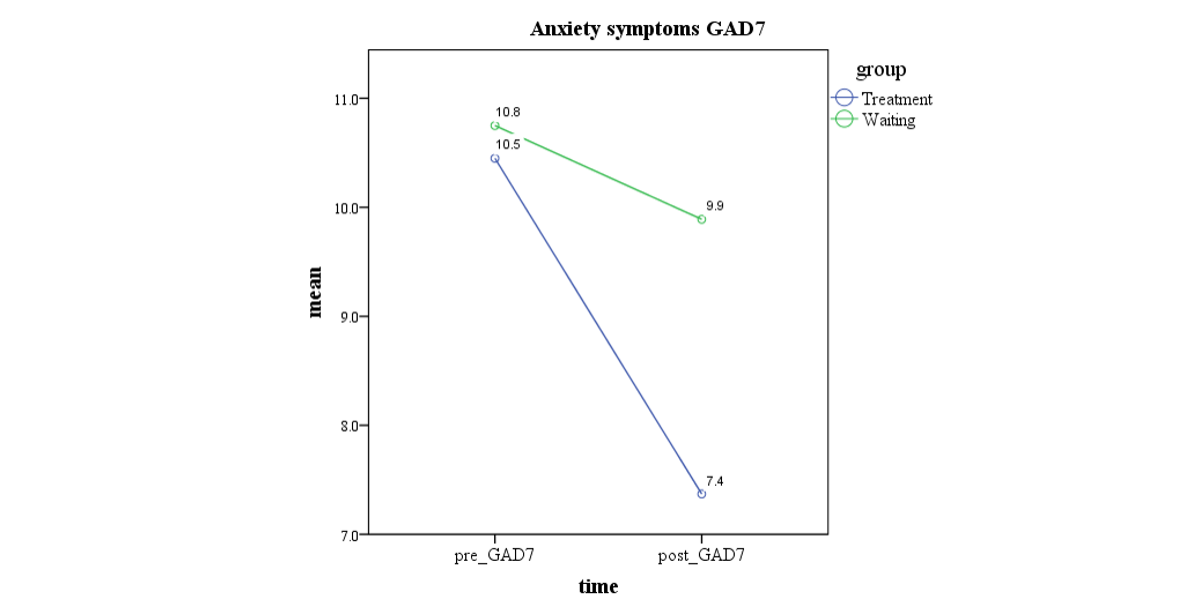
Changes in anxiety symptoms pre- to postintervention. GAD-7: Generalized Anxiety Disorder - 7.

The within-group results for the treatment group yielded a medium-to-large effect size after treatment (*d=*0.73; *P*≤.001), and this was maintained at the 3-month follow-up (*d=*0.52; *P*≤.001). The mean scores indicated that the treatment group reported significantly lower levels of anxiety symptoms after treatment than those in the WL control group, yielding a medium posttreatment effect size between the groups for the intervention (*d*=0.60; *P*≤.001). Figure 4 provides a graphical representation of the changes in anxiety symptoms between groups.

#### Clinically Significant and Reliable Change

RCI values were calculated in this sample population. For the PHQ-9, change was needed to exceed the RCI value of 3.97, and for the GAD-7, a value of 3.63, to be considered reliable. The proportion of each group that showed reliable improvements (or deterioration) between baseline and posttreatment and the 3-month follow-up was determined using these reliable change criteria. The analysis included only the number of participants who provided follow-up measures.

In the iCBT group, 76% (n=16) achieved reliable change, compared with 32% (n=17) in the WL control group. The differences between groups were statistically significant (X^2^_2_=10.519; *P=*.001). Reliable recovery from depression and anxiety was established by identifying the RCI and the percentage of participants who achieved a posttreatment score of ≤10 on the PHQ-9 and ≤8 on the GAD-7. In the iCBT group, 10 (n=10) participants met the criteria for reliable recovery on depression symptoms and 9 (n=9) met the criteria for the same on anxiety symptoms.

#### Satisfaction With Treatment

After 7 weeks, the participants (n=40) also completed the SAT questionnaire. Most of the users were happy to use a computer to access their treatment (76%, n=30) and found the interventions easy to use (82%, n=32). Furthermore, the majority of participants (68%, n=27) found the Web-based treatment helpful (Table 5).

**Table 5:** Results from the Satisfaction With Treatment questionnaire, (n=40).

Questionnaire items	Participants		
	Agree (%)	Disagree (%)	Neither (%)
I was happy to use the computer to access the treatment	76	8	16
I found the intervention easy to use	82	5	13
I feel the treatment received will have a lasting effect	63	8	29
I would recommend the Web-based treatment to others	68	16	16
How helpful you found the Web-based treatment program	68^a^	23^b^	10^c^

^a^Helpful.

^b^A little helpful.

^c^Not at all helpful.

The final two questions in the satisfaction measure were qualitative and asked participants what they most and least liked about the Web-based treatment. The majority of the users reported its accessibility and flexibility (n=20, 50%) as the aspects they liked the most. The other aspects liked were the applicability of the program to their life, having a supporter, and the interactive tools and the activities:

The availability of all the material that it includes and the sense of security that it gives to know that I can receive help at any time.

The strategies proposed are very effective that allows us to question about the [negative] habits have [are unhelpful] to lead a happier life.

...That I could keep track of my emotions and moods.

When asked what they *least liked* about the Web-based treatment program, some participants reported that the program did not meet their individual needs, such as immediate feedback or synchronic interaction with the supporter (n=11, 28%) and lack of time (n=4, 10%).

The platform is impersonal. Perhaps, it was not what I needed to hear. Perhaps, nothing replaces being in touch in real life with someone.

## Discussion

### Principal Findings

The study aimed at evaluating the efficacy of a culturally adapted *Yo puedo sentirme bien/I can feel better* program for college students with depressive symptoms in Colombia***.*** The results show a very high research attrition and treatment dropout in this sample. However, the treatment group demonstrated statistically significant decreases in depressive symptoms from baseline to posttreatment, relative to the pre-post change in the WL control group, and this reduction in symptoms was maintained at the 3-month follow up. Furthermore, a significantly larger proportion of the iCBT group achieved a reliable change, compared with the WL control group. The results from this study thus showed that the culturally adapted cognitive behavioral internet-delivered program, *Space from Depression*, is effective in reducing depressive symptoms in comparison with a WL control group. These results support previous research findings on the *Space from Depression* program in Ireland [59]. The findings presented here indicate that the program can potentially be used with other cultural groups, provided that it is modified to contain culturally adapted components.

The results in this study also showed that positive changes in depressive symptoms can be maintained by users at the 3-month follow-up. This is a relevant finding regarding maintenance of gains achieved from an internet-delivered intervention. Previous research [81,82] in culturally adapted internet interventions for depression with Arab and Chinese immigrants has been consistent in demonstrating lasting effects. For instance, a study [81] found that a reduction in depressive symptoms was still maintained at the 3-month follow-up within a population of Chinese immigrants.

Furthermore, this study found that the *Yo puedo sentirme bien*/*I can feel better* program is effective in reducing comorbid symptoms of anxiety in comparison with a WL control group. Statistically significant reductions were observed in anxiety symptoms for the intervention group from pre- to posttreatment, and these were maintained at the 3-month follow-up. The results support the efficacy of this internet-delivered cognitive behavioral intervention for depression and anxiety, as has been observed in a previous study of the intervention in English [59]. Similarly, a study [83] showed a significant reduction in depression and comorbid anxiety after treatment and at the 3-month follow-up in an RCT with Turkish immigrant residents in the Netherlands.

This RCT reports the results from the first culturally adapted internet-delivered program with asynchronous Web-based support in an LMIC in South America. The intervention showed similar outcomes to those established previously in Web-based treatments for depression and anxiety in HICs [51,59,84-86]. The evidence base for internet-delivered interventions for depression and anxiety symptoms comes mainly from people living in developed countries [56], whereas iCBT may be a valuable resource to reduce the treatment gap for those living in less developed countries, thus ameliorating social health inequalities between regions [58].

Furthermore, a reliable change was achieved by a greater percentage of those in the iCBT group (n=16, 76%), compared with those in the WL control group (n=17, 32%). The reliable change in internet-delivered interventions has showed greater percentage in different studies in the literature based on intention-to-treat (ITT) samples. For instance, a study of culturally adapted iCBT for depression reported a reliable change in the treatment group (32%) as measured using the Center for Epidemiological Studies-Depression [83]. In addition, a culturally adapted transdiagnostic iCBT for depression and anxiety [87] reported that participants made a 50% greater improvement on the PHQ-9 at posttreatment and the 3-month follow-up, respectively, using a posttreatment score at or below the clinical cutoff on the PHQ-9 (≥10) as a criterion for reliable change. A study of the internet-delivered treatment for depression upon which this cultural adaptation is based, the *Space from Depression* program, reported a 31% reliable change in the treatment group by using a criterion of change of ≥9 points on the Beck Depression Inventory-II from pre- to posttreatment [59].

The participants in the iCBT group spent an average of 3 hours and 18 min using the treatment and an average time of 18.2 min per session. Participants determined the amount of time they spent on the platform, which is a feature of internet-delivered interventions. A research study using the *Space from Depression* program with a sample from the general population in Ireland showed a greater mean time spent on the program in this population (5 hours 22 min) [59]. This difference in time spent using the intervention could be associated with time limitations within the college student sample in Colombia, where exam weeks and academic tasks can affect people’s use of the program. This time limitation among college students may have also compromised the success of the intervention (ie, the high dropout). Studies have identified factors that could influence the time spent on the Web, such as processing speed, cognitive ability, reading aptitude, and familiarity using computers [88].

#### High Levels of Depressive Symptoms and Suicidal Ideation

Both colleges reported high exclusion criteria at the beginning (64% of participants were excluded before they were randomized to treatment and WL control groups). For instance, in Bogota, a high proportion of students were excluded because of suicidal ideation (n=130, 24%) and drug/alcohol misuse (n=94, 17%). Similarly, in Bucaramanga, a high proportion of students reported suicidal ideation (n=23, 12%) and alcohol and drug misuse (18%, n=22). The exclusion might be considered higher compared with other studies in HICs [53,59,89]. In culturally adapted internet-delivered treatment for depression, high exclusion rates related to suicidal ideation have been reported in different populations [81,83]. Studies have supported that the reasons for the exclusion may include different interpretations that communities have about mental disorders that may not be considered in the measures used for depression [2,90]. More studies are needed in this area to develop validated measures considering cultural differences. In addition, college students typically report a higher prevalence of depression, suicidal ideation, and alcohol/drug misuse [20,28], and there are similar reports in South America [26,91].

Furthermore, the iCBT program was tested for mild-to-moderate depressive symptoms, which shows the necessity of its use in higher levels of depression or more precise measures to assess participants with more significant levels of depression, given how common it was for people to be screened out for psychological symptoms. The *Space from Depression* program has also reported efficacy with this population [92]; future studies should be considered at this point.

#### Attrition

##### Research Attrition

In this study, only 20% of the participants completed measures after treatment and 17% completed them at the 3-month follow-up. This very high attrition is not easily comparable with other culturally adapted internet-delivered treatment for depression studies. For instance, a study [83] showed that 58% participants completed the posttreatment measures (6 weeks), and 38% filled the follow-up assessment at 4 months. In addition, Choi et al [81] reported that 92% of the users completed the posttreatment questionnaires, and 84% of participants completed the questionnaires at the 3-month follow-up. Internet-delivered interventions for depression have showed higher retention rates, for example, a study [93] reported 49% of participants providing data at posttreatment and 35% at follow-up. In a previous study using the *Space from Depression* program, 63% of participants completed measures after treatment (8 weeks), and 52% completed 3-month follow-up measures [59]. Procedural variables could be associated with the very high research attrition rate in this study, such as phone numbers of the participants not being available from the beginning of the study and limiting phone calls to the participants to encourage them to complete the measures after treatment.

In this study, recruitment was conducted by email, and therefore, there was no face-to-face contact with the participants. Personal contact at the beginning of the intervention would have, perhaps, encouraged the participants’ engagement and continuation with the program. This is also associated with another factor linked to attrition—lack of reminders [94]. In this study, users received emails and calls at the end of the study to encourage them to complete the measures. Some limitations were present, such as the calls being made from Ireland, where there is a significant time difference with Colombia. Therefore, it was not possible to call the participants during a specific time that they might be more likely to answer the phone. Moreover, it is likely that for some participants, reminder emails went to spam.

Studies have suggested personality factors, cognitive abilities, and motivation as relevant determinants for attrition [95]. We did not evaluate these elements in this study. Future studies should examine strategies to decrease attrition rates, which is a determinant of effectiveness in internet interventions.

##### Treatment Dropout

Only a small proportion of participants completed all modules; 80% (86/107) of the participants in the iCBT group began with module 1, and 9% (10/107) completed all modules. Treatment dropout has been found to be a problem in internet interventions [94,96]. However, studies have shown that having a supporter is associated with higher engagement and efficacy in Web-based interventions [51,97]. Unfortunately, that was not the case in this study. Despite the training provided to the supporters in the Web-based program and their experience in CBT, the population engagement was low. The possible reasons could include barriers related to accessing mental health treatments in Colombia, such as lack of motivation for change, negative perception of psychological treatments, and personal stigma [26]. In addition, this is the first study with internet-delivered interventions in Colombia and in South America, which might affect the engagement with the treatment.

Furthermore, possible cultural differences and specific characteristics of the college student sample could be considered in future studies in Colombia to try to improve engagement with the treatment. For instance, the structure of the year and competing demands on students, for example, exams and holiday periods, can have a detrimental impact on engagement and adherence to any intervention. In addition, a lack of reminders has been associated with participant dropout [94]. In this study, participants received an automatic alert email each week from their supporter. It is probable that these automatic alerts were received as spam, and consequently, the participants could have forgotten to use the program and dropped out from the intervention. In addition, 87 participants (41%) of the population in this study reported low IT confidence, which could be another dropout factor and may affect engagement with the intervention [98]. Studies have associated the low use of technology with attrition in Web-based interventions [95]. Future studies could assess and develop strategies to support participants with low IT confidence. Activities could involve training and calling the participants to clarify or guide them on how to use the internet intervention. Despite the fact that the *Space from Depression* program involves interactive videos and information on how to explore the program, the supporter could also reinforce and guide the user with this as part of the weekly feedback. Considering these points, the analysis also revealed that participants who completed posttreatment measures showed higher program usage (mean number of logins 11, SD 7.32), compared with those who did not (mean number of logins 3.28, SD 2.76). These outcomes may be reflective of the low IT confidence reported by the sample.

In addition to this inexperience with IT, the intervention was implemented into a sample largely inexperienced in using counseling. Only 20% of the participants in this study had received psychotherapy previously, which could be considered another factor for dropping out. It also highlights the opportunity the study provided for these populations to have access to a treatment program for depression, showing the potential of iCBT to reach different, typically inaccessible, populations.

#### Satisfaction With Treatment

The results from the satisfaction and user-experience questionnaires indicated that, in general, participants were satisfied with accessing an iCBT intervention. However, a minority of participants felt the treatment did not meet their individual needs, as they found it difficult to get motivated and engage with the program. Flexibility and accessibility were most liked by the participants, which is consistent with previous studies [72,79]. Participants found the intervention easy to use and were happy to access the Web-based treatment. It should be noted that the SAT response rate was low and might be biased toward those who were satisfied with the program and found it helpful.

One of the most significant aspects reported by the participants was presence of a supporter. Having a supporter was valuable to encourage the user to continue with the program and provided guidance and feedback. Some participants reported feeling relieved because of the supporter or having a space to express themselves. All the participants in this study reported positive comments about their supporters, which may reflect the importance of having a trained postgraduate student as an appropriate component to provide suitable feedback to the users. However, a limited number of users share information with their supporter, reflecting a sense of anonymity unique to a Web-based treatment [99].

Participants reported aspects of time as something they least liked about the internet-delivered intervention, which was related to difficulties in completing all the modules in the program. Users reported a lack of time because of academic commitments or personal circumstances. Participants also reported that they did not have enough time to complete the modules in the program and feeling rushed to review the content between sessions. Similar findings have been reported previously [79].

### Strengths of the Research

First, the results show that the culturally adapted program *Yo puedo sentirme bien*/*I can feel better* was effective in reducing depressive symptoms and comorbid anxiety symptoms with college students in Colombia. The original *Space from Depression* program has been previously investigated with college students [100] and a community population [59], with significant improvement in depressive symptoms pre- to posttreatment in both populations. Most of the studies of internet-delivered interventions have been studied in HICs, which might not be generalizable to other populations worldwide. Therefore, knowing more of the effectiveness of internet interventions in LMICs may mean that this type of treatment can be an alternative for people who cannot access mental health services because of limited health insurance or personal stigma and is therefore relevant in Colombia [11,101].

Second, despite the high attrition rate found in this study, this type of treatment was an opportunity for students to have access to a psychological intervention. A recent report showed that only a small minority of college students receive adequate treatment for their mental disorders in low-income countries (6%), compared with HICs (23%) [28].

Third, this study adds supporting evidence for using trained student supporters in guided internet-delivered interventions for mild-to-moderate depressive symptoms. Several studies have reported that various levels and type of supporters can achieve positive outcomes in low-intensity internet-delivered interventions for depression [51,85]. Despite the high dropout rate in this study, the student supporters in the study might play an important role in increasing engagement with the program. Corroborating this, qualitative comments by the participants on the SAT were positive about the interaction with their supporters.

Finally, this type of intervention could be delivered through a counseling service with trained students in Colombia. Similarly, it may be employed as a potential alternative in primary care or other populations. Studies have evidenced that internet-delivered interventions might potentially reduce the barriers to treatment access in LMICs [30]. In addition, culturally adapted internet-delivered treatments could reduce cost and possibly personal stigma [102]. Therefore, the study alludes to the potential of a culturally adapted internet-delivered intervention, with support, to accomplish significant outcomes. In locations where mental health services are underdeveloped, health care structures do not exist, or there is a potential to offset risk and escalation of difficulties and benefit from early intervention, such a model of service provision could be feasible [103].

### Limitations

A noted limitation is that the study did not include an official depression diagnosis of participants; however, it includes well-established measures of symptom severity that can allow us to establish the efficacy of low-intensity internet-delivered treatments for depression symptoms among college students in Colombia. Another concern is with regard to the problem of missing data.

Owing to the high attrition of research data, it would not have made sense to complete a true ITT analysis including appropriate imputation of missing values. Therefore, our ITT analysis is not as robust as we would have liked, and as a result, caution is advised in any interpretation of the results from this study.

The research attrition was high at posttreatment, and it was even higher at the follow-up. Reminders in the form of emails were sent (maximum four times) and phone calls were made with some participants who provided their contact details (maximum three times), but this did not result in a significant decrease in study dropout/attrition rate. The reasons for this high attrition rate are not entirely known. In addition, the cultural adaptation of the program was not further evaluated after the intervention with the participants, which might have allowed us to assess and identify potential factors related to the adaptation that could have affected dropout.

### Future Research

Internet-delivered interventions for depression have shown significant effects post treatment [51], specifically, in high-income Western countries. LMICs around the world have scarce access to mental health services [26]. A strategy to utilize the internet to provide more widely available and low-cost mental health care has vast potential. In addition, internet-delivered treatments could contribute to the globalization of mental health services and psychological interventions, for which cultural adaptation is the key [33].

This study was the first contribution regarding the potential impact of a culturally adapted internet-delivered, low-intensity intervention on depressive symptoms for college students, as compared with a WL control group, in South America. Internet-delivered interventions could be a potential option for delivering evidence-based psychological interventions and may overcome some of the significant barriers to accessing mental health treatment in Colombia. Future research should focus on monitoring participants who drop out prematurely from the study at a follow-up to evaluate the reasons for withdrawal.

This study has demonstrated the potential for internet-delivered interventions to provide satisfactory, acceptable, and effective low-intensity treatments to individuals living with depression in South America. The accessibility and flexibility unique to a Web environment may increase the access to mental health interventions around the world, considering the cultural characteristics of the populations, and this may contribute to decreasing the mental health treatment gap in LMICs.

In future studies, analysis of the central characteristics of the sample, such as age, previous level of depression, familiarity with the technology, previous treatments, and the perceived usefulness of the treatment, among others, could show the extent to which different groups of people might benefit more from this type of Web-based treatment. In attempting to explain and understand the reasons for dropout in the sample, a number of reasons were speculated upon. However, future studies might analyze further the population’s previous knowledge about depression, any associated personal stigma, and their perception of how psychological treatment could help overcome it.

### Conclusions

This study showed that the culturally adapted iCBT program *Yo puedo sentirme bien*/*I can feel better* could be employed in different cultural groups as a population-level intervention for mild-to-moderate symptoms of depression. Importantly, outcomes from the treatment group were maintained at the follow-up. The research attrition was high at posttreatment and at the follow-up in this study. Future research should focus on monitoring participants who drop out prematurely from the study at a follow-up to evaluate the reasons for withdrawal. The results from this study are encouraging for the implementation of the culturally adapted internet-delivered cognitive behavioral program, *Space from Depression*, for the treatment of depression symptoms in a culturally different population, helping disseminate evidence-based treatments in LMICs.

## Appendix

Multimedia Appendix 1 Screenshot of the Space from Depression program.

Multimedia Appendix 2 Tools - Space from Depression program.

Multimedia Appendix 3 CONSORT-EHEALTH checklist (V 1.6.1).

## Abbreviations

CBT: cognitive behavioral therapy
CONSORT: Consolidated Standards of Reporting Trials
CRQ: Cultural Relevance Questionnaire
DSM IV: Diagnostic and Statistical Manual of Mental Disorders, fourth edition
GAD-7: Generalized Anxiety Disorder - 7
HIC: high-income country
iCBT: internet-delivered cognitive behavioral therapy
IRC: Irish Research Council
IT: information technology
ITT: intention-to-treat
LMIC: low- and middle-income country
LMM: linear mixed model
PHQ-9: Patient Health Questionnaire - 9
RCI: reliable change index
RCT: randomized controlled trial
REML: restricted maximum likelihood
SAT: Satisfaction With Treatment
WL: waiting list
