# Supplementary figures and images for: A Culturally Adapted Cognitive Behavioral Internet-Delivered Intervention for Depressive Symptoms: Randomized Controlled Trial
Source: JMIR Ment Health. 2020 Jan 31;7(1):e13392. doi: 10.2196/13392 (PMC7055858; doi:10.2196/13392)

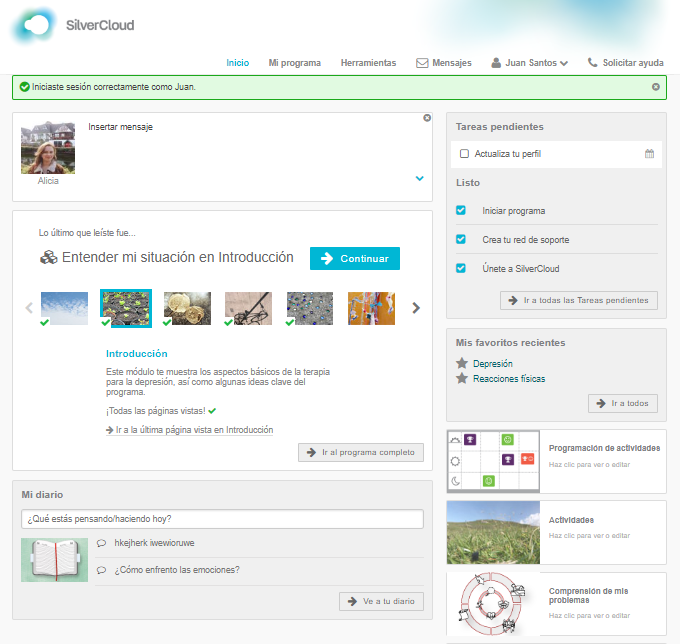

Supplement: Multimedia Appendix 1 [file mental_v7i1e13392_app1.png]

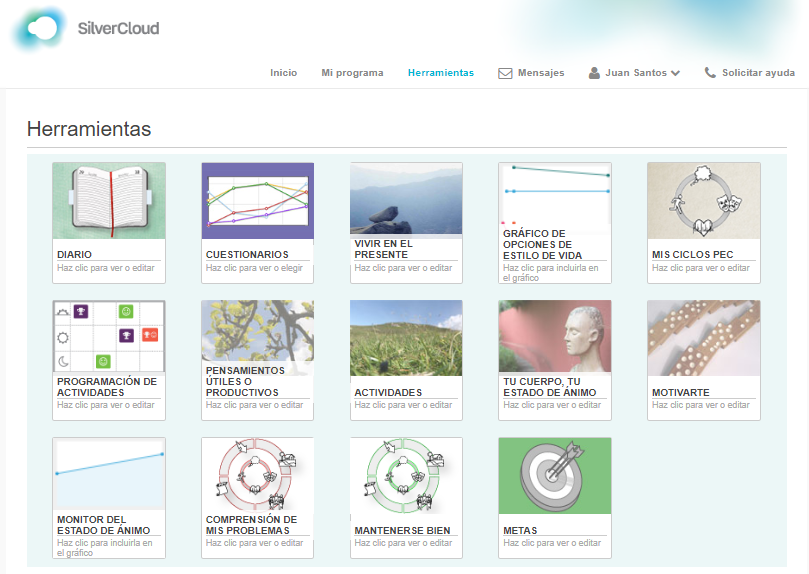

Supplement: Multimedia Appendix 2 [file mental_v7i1e13392_app2.png]
